# Editorial: Intracellular Mechanisms of α-Synuclein Processing

**DOI:** 10.3389/fcell.2021.752378

**Published:** 2021-09-14

**Authors:** Friederike Zunke, Beate Winner, Franziska Richter, Gabriela Caraveo

**Affiliations:** ^1^Department of Molecular Neurology, University Hospital Erlangen, Friedrich-Alexander-Universität Erlangen-Nürnberg, Erlangen, Germany; ^2^Department of Stem Cell Biology, Friedrich-Alexander-Universität Erlangen-Nürnberg, Erlangen, Germany; ^3^Department of Pharmacology, Toxicology, and Pharmacy, University of Veterinary Medicine Hannover, Hanover, Germany; ^4^Department of Neurology, Feinberg School of Medicine, Northwestern University, Chicago, IL, United States

**Keywords:** alpha-synuclein, protein misfolding, lysosomes, spread, neurodegeneration, synucleinopathies

The aggregation of the protein α-synuclein (aSyn) is the pathological hallmark of the group of neurodegenerative disorders, collectively known as synucleinopathies. These include Parkinson's disease (PD), PD-Dementia, Dementia with Lewy Bodies (DLB), and Multiple Systems Atrophy (MSA). While all of these neurodegenerative disorders present with distinctive clinical features, they all converge in one pathological characteristic: intracellular aSyn aggregation into Lewy Bodies (Mezey et al., 1998; Spillantini et al., 1998; Goedert et al., 2017; Riederer et al., 2019). Lewy Body pathology can occur at the soma and neurites of neurons, but it can also occur within glial cells as in MSA [called glial cytoplasmic inclusions (GCI)]. To complicate matters, there is increasing evidence for extracellular aSyn conformers, that might be responsible for the spreading of pathological protein aggregates and hence disease pathology (Kordower et al., 2008; Li et al., 2008), as first demonstrated in patients following fetal midbrain transplants. This findings have led to the hypothesis that sporadic PD might progress in six states that follow a caudo-rostral pattern (Braak et al., 2003), with peripheral non-motor symptoms occurring before the diagnosis of the full blown disease. Despite the central role of aSyn in all of these disorders, little is known about the initial mechanisms that lead to its aggregation, disruption of cellular functions and extracellular spread, as suggested via the gut-brain axis (Kim et al., 2019; Derkinderen et al., 2020). Articles within this Research Topic seek to shed light into these mechanisms.

aSyn is typically degraded by both the lysosome and the proteasome (Cuervo et al., 2004; Shin et al., 2005). It is of no surprise that mutations in genes associated with lysosomal pathways are major genetic risk factors for the development of PD (Klein and Mazzulli, 2018). These include the lysosomal enyzmes β-glucocerebrosidase (GBA1), galactocerebrosidase (GALC), and the lysosomal cathepsins (CTSD and CTSB), as well as lysosomal membrane proteins like SCARB2, TMEM175, LAMP3, and components of the lysosomal acidification machinery (ATP13A2 and ATP6V0A1) (Sidransky et al., 2009; Chang et al., 2017; Robak et al., 2017). As shown in longitudinal studies, GBA1-associated PD patients undergo faster disease progression and shorter survival, underlying the need for novel and genotype-specific therapeutic strategies (Brockmann).

GBA1 degrades the lysosomal sphingolipid glucosylceramide into glucose and ceramide. Mutations in GBA1 linked to PD, yield deficits in ceramide metabolism and result in inefficient aSyn degradation within the lysosome. Accumulation of the GBA1 substrate, glucosylceramide can lead to the conversion of physiologic to pathologic aSyn (Zunke et al., 2018), indicating lipids as one of the key factors in aSyn conformation (Kiechle et al.) (Figure 1, no. 1, 5).

**Figure 1:**
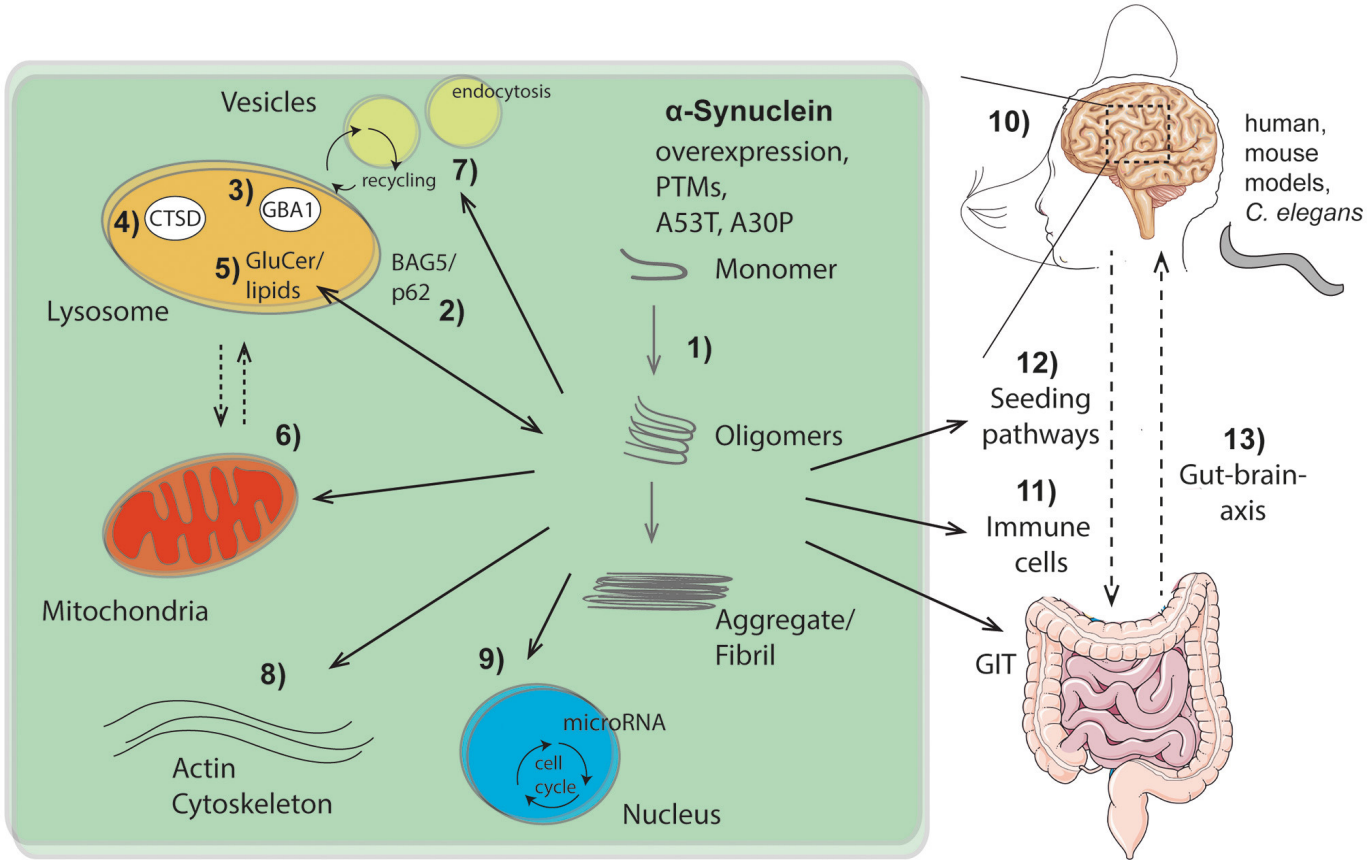
Overview of intra- and extracellular routes of aSyn aggregation and pathology pathways as highlighted within this Research Topic: (1) Intracellular aSyn aggregation can be triggered by overexpression, post translational modifications (PTMs), or mutations within aSyn (e.g., A53T, A30P). (2–5) Pathological aSyn conformers comprising oligomers and fibrils block the autophagic/lysosomal pathway by interfering with BAG5 and the autophagic adaptor protein p62 (2), the lysosomal enzymes β-glucocerebrosidase (GBA1; 3) and cathepsin D (CTSD; 4), all critical for aSyn degradation. Dysfunction of GBA1 causes glycosphingolipids (glucosylceramide, GluCer) to increase (5). These lipids further drive aSyn aggregation. Pathological aSyn conformers also affect mitochondrial function, the lysosomal-mitochondrial crosstalk (6), vesicle recycling, and endocytosis (7), as well as formation and function of the actin cytoskeleton (8). Moreover, aSyn accumulation induces microRNAs involved in cell cycle activation (9). (10) Effects of aSyn-mediated pathologies were analyzed and summarized within different models (human, murine, *C. elegans*), exhibiting important roles of aSyn within the hippocampus. Additionally, aSyn is capable of escaping neurons causing cell-to-cell propagation and hence spreading of disease, which causes pathological effects on peripheral immune cells (11) and the gastro intestinal tract (GIT). The gut-brain-axis contributes to the spread of pathological aSyn conformers and disease pathology (12, 13). This illustration contains images from Servier Medical Art (smart.servier.com).

Further emphasizing the importance of lysosomal degradation processes in synucleinopathies (Figure 1, no. 2-5), as well as the bidirectional loop between degradative function of lysosomes and aSyn proteoforms (Wildburger et al.), lysosomal cathepsin D variants associated with neurodegenerative disorders were analyzed (Bunk et al.) (Figure 1, no. 4). Given that lysosomal cathepsins have been shown to directly process aSyn (Mcglinchey and Lee, 2015), the study of Bunk et al. also suggests enhanced aSyn proteolysis as a potential therapeutic strategy. Since the lysosome is the key organelle involved in autophagy, defects in autophagic function have been implicated in numerous neurodegenerative diseases including synucleinopathies. Highlighting the link between lysosomal autophagic pathways and aSyn accumulation, Friesen et al. describe that the co-chaperone BAG5 can promote aSyn oligomer formation, as well as regulate the levels and subcellular distribution of p62, an important autophagic adaptor protein (Friesen et al.) (Figure 1, no. 2).

The structural properties and posttranslational modifications (PTMs) of aSyn play an important role in toxicity and its seeding capacity (Figure 1, no. 1, 12). To this end, Ray et al. revises the importance of aSyn structure and mutations on the biophysics of its aggregation, cell autonomous pathobiology, as well as spreading of disease (Ray et al.). Consequences of two common familial-associated mutations (A30P and A53T) were evaluated on protein aggregation and locomotor behavior in a *C. elegans* model (Perni et al.). Furthermore, Fouka et al. summarizes potential treatment strategies aiming at preventing both protein aggregation and cell-to-cell propagation via utilization of antibodies against aSyn (Fouka et al.). Moreover, lysosomal as well as mitochondrial pathways are highlighted for therapeutic strategies via calcium and iron modulation among others (Minakaki et al.).

Once in the pathogenic form (Figure 1, no. 1), aSyn can lead to several cellular and functional defects. These cellular deficits range from epigenetic changes through an induction of microRNAs involved in cell cycle activation with deleterious consequences for dopaminergic neurons (Findeiss et al.) (Figure 1, no. 9), to changes directly affecting synapse dynamics. These include changes in vesicle recycling (Soll et al.) (Figure 1, no. 7), as well as defects in the actin cytoskeleton (Oliveira da Silva and Liz) (Figure 1, no. 8). Along the lines of synaptic perturbances caused by aSyn are the findings of Regensburger et al. Using a transgenic mouse model of pathogenic aSyn, they find impaired postsynaptic integration of adult hippocampal newborn neurons, underlining the role of postsynaptic degeneration as an early feature in synucleinopathies (Regensburger et al.). Reinforcing the role of aSyn in the adult hippocampus, an increase in the number of early stage neuronal progenitors in a human aSyn transgenic mouse model was shown (Bender et al.) (Figure 1, no. 10). These studies uncover novel aspects of aSyn pathology in adult neurogenesis and suggest a mechanism that might explain the early cognitive deficits observed in both DLB and PD-dementia (Aarsland, 2016).

Finally, the aggregation properties aSyn and cellular defects are not locally confined, but appear to be global too. Altered immune cell phenotypes have been reported in aSyn animal models as well as in human disease (Cao et al., 2011; Grozdanov and Danzer) (Figure 1, no. 11). Recently, a strong association between clinical manifestations within the gastrointestinal tract (GIT) and PD has been described (Schaeffer et al.). A better comprehension of aSyn function and structure within the GIT will be crucial to understand its role in the enteric nervous system and its role in spreading from the gut to the brain (Figure 1, no. 12, 13).

In summary, the articles within this Research Topic provide an overview of intracellular mechanisms that mediate the conversion from physiologic to toxic aSyn conformations, the intracellular consequences of toxic aSyn, as well as spreading mechanisms that accelerate pathology in nearby cells and other tissues (Figure 1). A better understanding of the pathological events leading to synucleinopathies will be critical to design targeted therapeutic strategies to combat these devastating neurodegenerative disorders for which no cures exist yet.

## Author Contributions

FZ provided the figure. All authors contributed to the manuscript.

## Funding

FZ is supported by the German Research Foundation (DFG), grant number 125440785 - SFB 877 (project B11) and the Interdisciplinary Center for Clinical Research (IZKF) at the University Hospital of the University of Erlangen-Nuremberg (Jochen-Kalden funding programme N8). Additional support for BW came from the Bavarian Ministry of Science and the Arts in the framework of the ForInter network, the German Research Foundation, DFG WI 3567/2-1 and 270949263/GRK2162, and the IZKF advanced project E30. GC is supported by the Parkinson's Foundation PF-JFA-1949 and R01 NS117750/NS/NINDS NIH HHS/United States.

## Conflict of Interest

The authors declare that the research was conducted in the absence of any commercial or financial relationships that could be construed as a potential conflict of interest.

## Publisher's Note

All claims expressed in this article are solely those of the authors and do not necessarily represent those of their affiliated organizations, or those of the publisher, the editors and the reviewers. Any product that may be evaluated in this article, or claim that may be made by its manufacturer, is not guaranteed or endorsed by the publisher.
